# Digital Sun Sensor Multi-Spot Operation

**DOI:** 10.3390/s121216451

**Published:** 2012-11-28

**Authors:** Giancarlo Rufino, Michele Grassi

**Affiliations:** Department of Aerospace Engineering, University of Naples “Federico II”, Piazzale Tecchio 80, 80125 Naples, Italy; E-Mail: giancarlo.rufino@unina.it

**Keywords:** sun sensor, APS, centroiding, attitude determination, image processing

## Abstract

The operation and test of a multi-spot digital sun sensor for precise sun-line determination is described. The image forming system consists of an opaque mask with multiple pinhole apertures producing multiple, simultaneous, spot-like images of the sun on the focal plane. The sun-line precision can be improved by averaging multiple simultaneous measures. Nevertheless, the sensor operation on a wide field of view requires acquiring and processing images in which the number of sun spots and the related intensity level are largely variable. To this end, a reliable and robust image acquisition procedure based on a variable shutter time has been considered as well as a calibration function exploiting also the knowledge of the sun-spot array size. Main focus of the present paper is the experimental validation of the wide field of view operation of the sensor by using a sensor prototype and a laboratory test facility. Results demonstrate that it is possible to keep high measurement precision also for large off-boresight angles.

## 1. Introduction

Sun sensors are typically used to provide coarse-to-medium accuracy measurements of the sun line in the satellite-fixed axes. This information is essential for autonomous attitude determination in the various phases of a space mission [1]. In the recent years, many space missions based on microsatellites and nanosatellites have been conceived. This class of platforms offers several advantages in terms of mission time scale and flexibility, but, at the same time, it requires the design of ever more compact and miniaturized components and units.

In recent years, a new generation of attitude sensors, e.g., star and sun sensors, has emerged which relies on imaging devices [2,3]. Concerning sun sensors, they adopt linear or planar CCD or Active Pixel Sensors (APS) as focal plane detectors and a mask placed on the top at a certain distance. The mask has tiny slits or pinhole apertures to produce sun images on the focal plane from which the sun-line direction can be extracted. These sensors offer medium-to-high measurement accuracy, depending on the optical head design and the algorithms used to process the sun images [4–8].

Based on this scenario, within a program sponsored by the Italian Space Agency (ASI) to fly a number of innovative technology payloads on board the first Italian microsatellite platform MIOsat [9,10], the prototype of a two-axis Micro Sun Sensor (MSS) has been developed and tested at the Department of Aerospace Engineering (DIAS) of the University of Naples [11–18]. By exploiting a multi-aperture mask design, this APS-based sensor provides sun-line measurements with high accuracy and precision over a restricted Field Of View (FOV). With respect to previous studies [4], additional features of the sensor under development are [12]:

- Use of neural calibration function;
- Use of COTS (Commercial off-the-Shelf) components and units

As described in detail in the following sections, the optical head adopts an opaque mask with 100 holes, so to produce many simultaneous images of the sun on the focal plane. Once processed, they can be averaged to improve sun-line precision. Extensive test campaigns have been conducted in previous papers [12–14,16–18] to:

- calibrate the sensor
- validate sensor operation with 100 spots (*i.e.*, on a restricted FOV)
- investigate the attainable performance in comparison to the theoretical limit
- check the effectiveness of the implemented software routine in laboratory-reproduced in-orbit experiments.

With respect to previous studies [4], the MSS is designed to also be operated with a variable number of sun spots, to allow operation on a wider FOV. Even though in previous author’s works (e.g., in [12]) the sensor operation on a wider FOV has been already investigated at a preliminary stage, more recent studies have been particularly focused on developing techniques, procedures and algorithms more suitable for this operation mode. Indeed, operation with multiple apertures implies that the number of acquired spots reduces with the increasing off-boresight angle. In addition, the degraded quality of the sun images acquired at large off-boresight angles may cause the image acquisition and processing procedure to fail in the identification of the correct number of sun spots on the focal plane. This degrades sensor angular precision performance.

In this paper these aspects are analyzed in detail and the adopted solutions are presented. The sensor wide-FOV operation is validated by using the available hardware model of the sensor and a dedicated test facility, in which in-orbit illumination conditions and variable sun-line orientation can be reproduced.

## 2. Multi-Spot Sensor Concept

**Figure 1.**
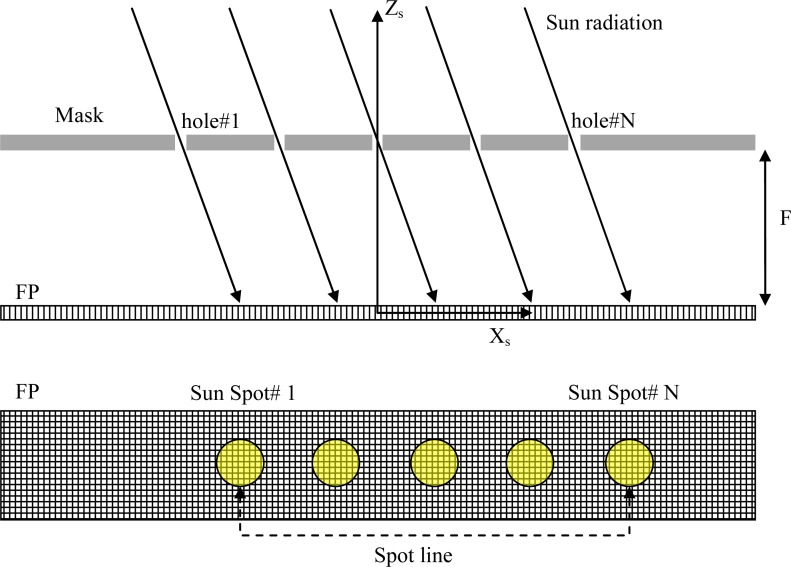
Sensor multi-spot operation concept (example of linear array of mask apertures for the sake of clarity).

As illustrated in Figure 1, the sensor image forming system consists of an opaque mask with multiple pin-hole apertures producing multiple simultaneous sun images (bright spots) on the Focal Plane (FP). The image pattern replicates the pattern of the apertures in the mask. This pattern moves on the FP as the illumination direction varies in the Sensor-fixed Reference Frame (SRF), with axes X_s_,Y_s_,Z_s_. The advantage of having multiple apertures is that basing the sun-line estimate on *N* spots, each of which can generate an independent measure, theoretically produces a precision improvement by a factor 1/N^1/2^[12].

The sun-line orientation is observed as the position on the FP (computed by proper image processing techniques [12,15]) of the average centroid of the array of sun spots. This procedure allows reducing the computational load so to perform multi-spot sun-line determination with adequate update frequency [16]. A neural calibration function is used to transform the average centroid coordinates into the sun-line orientation [12].

The sensor prototype exploits a mask with 100 holes arranged as a 10 × 10 array. Mask, photodetector and FP electronics compose the sensor Optical Head (OH). The sensor is completed by a CPU in charge of image processing and sun-line computation. This last one is an AMD-Geode-LX-based single-board computer in pc-104 format by RTD™. It is a COTS product designed for operation in harsh environment (*i.e.*, extended temperature, conduction-based cooling). It includes all the needed peripherals and interface in a single board. It is also equipped with a pc-104 power-conditioning module to regulate the power input from the unregulated bus and to supply both the CPU and the OH. The latter one is powered by the CPU at 5 Vdc via the USB link, which is also used for CPU-OH data exchange. OH and CPU are distinct functional units as well as distinct physical units. More details on the sensor prototype are available in [12]. For reader convenience, Table 1 summarizes the main technical characteristics of the sensor model developed at the laboratory of the University of Naples in view of its installation on the MIOSAT platform.

**Figure 2.**
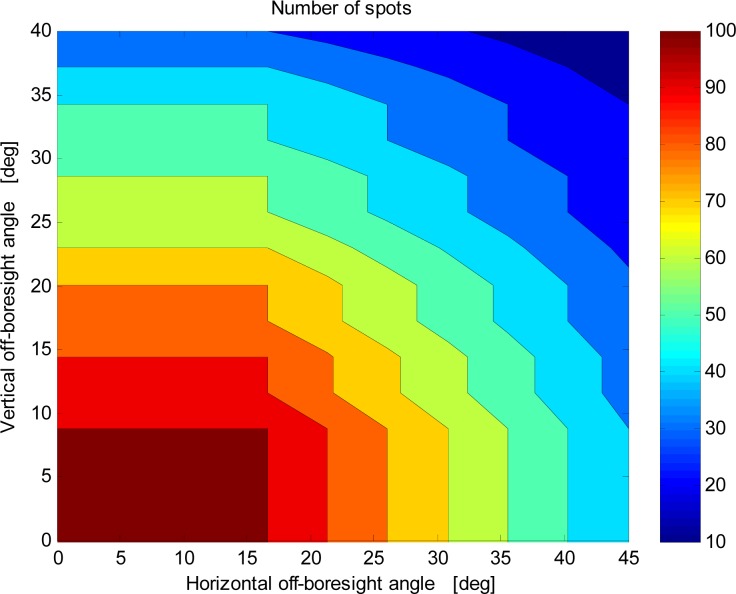
Total number of imaged sun spots *versus* the sun-line orientation in the sensor FOV: due to symmetry, only data for one FOV quadrant are shown.

The available FOV depends on the sensor operation mode. More specifically, the sensor is designed to be operated both with a fixed number of spots (Fixed-Spot Mode) and with a variable number of spots (Variable-Spot Mode). In the first case, the sensor operation always relies on detecting 100 spots, that are processed to compute the sun line with the best achievable precision. Fixed-spot operation and related performance are documented in detail in previous papers [12,16]. In 100-spot operation the available FOV is limited to about ±17° × ±8° since out of this region the 10 × 10 spot array is not fully imaged within the useful area of the focal plane (*i.e.*, the photodetector sensing surface). In this regard, Figure 2 shows the total number of imaged spots as a function of the sun-line orientation in the FOV: to better visualize the general trend only part of the available data set is shown (one quadrant of the FOV).

## 3. Wide FOV Sensor Operation

**Figure 3.**
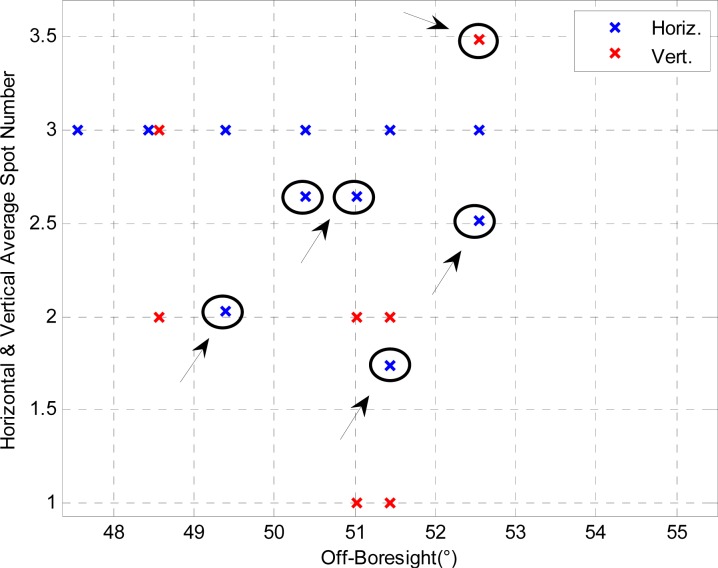
Variations in spot array size identification for large off-boresight angles.

The sensor wide-FOV operation, in the following referred to also as eXtended FOV (X-FOV) mode, cannot be performed with the same image acquisition procedure used for the fixed-spot operation. Indeed, in fixed-spot mode the sensor OH is operated with a fixed shutter time, computed so that the intensity of the sun spot pixels is in the order of 70% of the saturation level with the sun at the sensor boresight [13]. Preliminary laboratory tests of the sensor wide-FOV operation showed that with fixed-shutter operation the image quality degrades for increasing off-boresight angles. The image Signal-to-Noise Ratio (SNR) reduces since a lower amount of radiant energy is collected at the sensor entrance aperture. In addition, it is distributed over a larger sensing area on the FP. As a result, the software routine in charge of bright spot detection and centroid computation may fail in the identification of the correct number of spots imaged on the focal plane. A line (a row or a column) of the spot array close to FOV border may be not identified, even though all the spots of the line are completely imaged, since they are too faint with respect to the image background. This random event causes the variation of the acquired spot array size during repeated measurements at a given off-boresight illumination direction. This effect is displayed in Figure 3, which shows the average number of horizontal (in blue) and vertical (in red) spots acquired for some off-boresight directions. As a result, the sun-line computation cannot be carried out reliably, since the failure in detecting the correct size of the array of imaged spots causes position shifts of the average centroid much larger than the fluctuations observed when the same array size is processed.

Laboratory tests also showed that spot identification failures almost always happen when at least one of the array sizes is less than 4. In other words, only 4 × 4 or larger sun spot arrays can be processed reliably. This limits sensor operation to the acquisition of at least 4 × 4 spots, thus reducing the actual X-FOV size to about ±40° × ±35°. Moreover, when an array of spots of size 4 × 4 or slightly larger is imaged near the FOV border spot identification failures occur with a frequency of one case out of 50.

### 3.1. Image Acquisition Procedure Modification

To get reliable operation over a wider FOV, the image acquisition procedure has been modified with respect to one used for fixed-spot operation by introducing a variable shutter time. Before sun-line measurement a preliminary image acquisition is carried out to measure the sun spot intensity level. If it is under a preset threshold, the shutter time is automatically increased by exploiting the following simplified linear relation:

(1) $$\Delta t_{\mathrm{adj}}=\Delta t_{\mathrm{meas}}\frac{I_{\mathrm{target}}}{I_{\mathrm{meas}}}$$

where *Δt_meas_* and *I_meas_* are, respectively, the shutter time value to be modified and the sun spot pixel level before shutter time modification, *I_target_* is the desired intensity value, set to 70% of pixel saturation level. The resulting spot brightness is checked, and, if necessary, the shutter time is iteratively modified till convergence within 10% of the target value. Although more complex adjustment algorithms could be exploited, the use of a simple linear one is justified by the need of operating the sensor on board.

As shown in the following section, the shutter time adjustment allows increasing the SNR of images acquired in off-boresight conditions with respect to the same images acquired with a fixed shutter time. The improvement is higher with increasing off-boresight angles, thus making the process of sun-line determination much more reliable and precise.

In addition to operation with a variable shutter time, a different setting of some photodetector parameters has been introduced to improve further acquired image quality. More specifically, on-chip image processing for black level calibration (*i.e.*, background noise level evaluation and pixel correction) has been overridden, deferring it at the stage of the image processing during sun spot detection [15]. For the sake of conciseness, in the following the fixed and adaptive shutter procedures are referred to as basic and enhanced image acquisition modes.

### 3.2. Neural Calibration

Once the average centroid coordinates on the FP have been determined, they are transformed into the sun-line α and β angles, respectively the horizontal and vertical off-boresight angles of the sun line in the sensor FOV, by means of a neural Calibration Function (CF) [12,16]. Indeed, since the transformation from centroid coordinates to sun-line orientation is a highly non-linear, two-dimensional problem, using Neural Networks (NNs) provides a viable and effective solution to achieve high measurement accuracy without introducing complex geometrical models [12]. In addition, NNs with supervised training are universal approximators, *i.e.*, they can approximate to any desired degree of accuracy any real-valued, continuous function (or *sufficiently regular* function, with a countable number of discontinuities between two compact sets). Finally, using Neural Networks allows implementing the required mapping function without any prior assumption about the centroid-to-sun-line transformation, since the non-linear mapping can be built on the basis of experimental data only.

**Figure 4.**
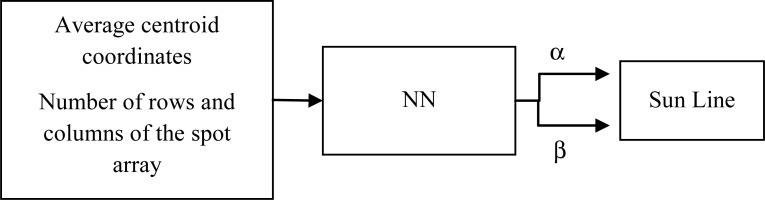
NN schematic.

A multilayer feed-forward NN with sigmoid activation function in the hidden layer and linear output neurons is considered for this application. More specifically, the selected NN structure consists of one hidden layer with twenty neurons and one output layer with two neurons. This NN architecture has been identified as the best one after several tests in which NN architectures characterized by a different number of neurons in the hidden layer have been compared. Indeed, the most suitable number of neurons in the hidden layer cannot be uniquely fixed, but it is peculiar to the application at hand, and has to be determined during NN training and validation. As shown in Figure 4, one single NN instead of two independent NNs [12], one for each angle, is used to transform the average centroid coordinated into the two angles defining the sun-line orientation in SRF. This allows reflecting into the NN structure the dependence of the average centroid coordinates on both rotations, that would result also from the application of the most simple geometrical model [12]. NN training, validation and test have been performed by using standard tools in Matlab environment [19]. Performance in NN training and test are shown in the next section. It is worth outlining that the adopted NN must receive in input not only the coordinates of the average centroid but also the number of rows and columns of the imaged spot array to operate in the X-FOV mode.

## 4. Wide FOV Operation Validation

### 4.1. Test Campaign

A test campaign has been conducted within the laboratory test facility described in [12–14,16–18] with the objective of validating the sensor operation in the X-FOV mode. Specifically, 483 uniformly-spaced illumination directions in the sensor X-FOV arranged in a 21-row × 23-column grid covering the angular region ±45° × ±40° have been considered. Nearly100 directions out of the latter set, randomly selected so to cover the entire X-FOV, have been used to test NN performance in sun-line measurement. The rest of the samples have been used for NN training and validation. For each reproduced illumination direction 70 images have been acquired with both fixed and adaptive shutter time.

### 4.2. Image Processing Procedure Validation

To get a quantitative estimate of the improvement achieved with the enhanced procedure, a comparison with the basic procedure has been performed in terms of the Signal-to-Noise Ratio (SNR) of the acquired images. Specifically, the SNR has been computed as:

(2) $$\mathrm{SNR}=\frac{I_{\mathrm{spot}}}{I_{\mathrm{back}}}$$

where *I_spot_* is the average intensity of the brightest imaged sun spot, and *I_back_* is the average intensity of the image background, *i.e.*, the area without sun spots.

**Figure 5.**
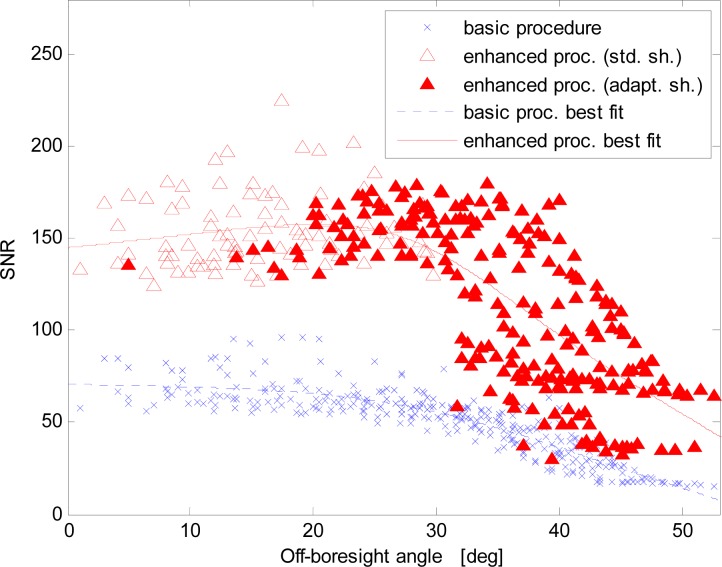
SNR *versus* the off-boresight angle for the basic and enhanced procedures.

**Figure 6.**
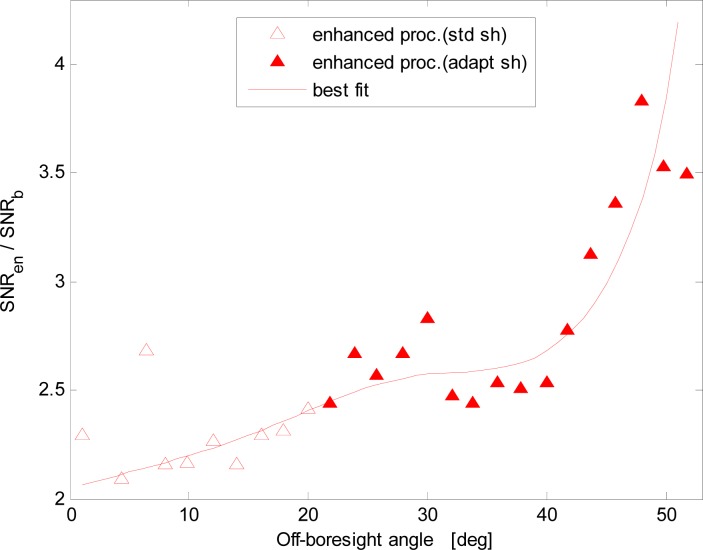
Ratio between the SNRs for the basic and enhanced procedures.

A comparison of the SNRs of the two procedures as a function of the increasing off-boresight angle is shown in Figures 5 and 6. More specifically, Figure 6 shows the ratio of the *SNR* of the same images acquired with the enhanced and basic procedures. To improve figure clarity, the plotted data samples are averages over 2-deg intervals of the off-boresight angle. The sample points of the curve in Figure 6 have been computed as the ratio of the sample points of the best fit curves in Figure 5 for the same value of the off-boresight angle. Several issues are evident:

- the image *SNR* is very well correlated to the distance from the sensor boresight, which confirms that viewing/imaging geometry definitely affects the quality of the images. This effect is low for limited off-boresight angles, but the *SNR* decays almost exponentially at large off-boresight angles;
- with the new procedure the SNR significantly improves, both near the boresight, due to the different solution adopted for the background noise threshold estimation, and at large off-boresight angles where the adaptive-shutter time operation is performed;
- near the boresight only the different black-level calibration is operated;
- starting from an off-boresight angle of about 25°, the shutter time adaptation is automatically activated;
- the SNR of the enhanced procedure exhibits a maximum value around 25° determined by the fact that the shutter time adaptation effect starts adding to the effect of the black-level calibration removal. Then, with increasing off-boresight angles the SNR starts rapidly reducing for both procedures;
- in terms of *SNR*, the improvement ranges from about 2.2 times at boresight to about 4.8 times at the largest off-boresight angle considered here (about 53 deg, at the corner of the FOV).

It is worth noting that in the enhanced mode the SNR values result more scattered. This is due to the adopted shutter-time adjustment algorithm that is based on using only a lower threshold for the average spot intensity and a 10% margin for the adaptation of the actual intensity level to the desired one. A refinement of the algorithm would reduce the variability.

**Figure 7.**
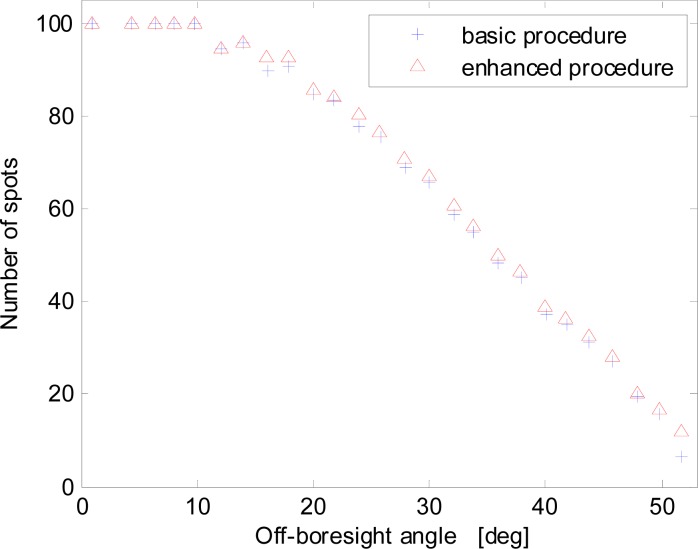
Average number of acquired sun spots *versus* the off-boresight angle for the basic and enhanced procedures.

These results are confirmed also by the analysis of Figure 7, which demonstrates that the number of spots acquired with the enhanced procedure is almost always higher for increasing off-boresight angles. This also improves sensor precision at large off-boresight angles. For a better description of the spot number trend Figure 7 has been produced by using only part of the available data set.

### 4.3. Measurement Performance Analysis

The data set used to build the NN has been divided in three sub-sets for NN training (70%), validation (15%) and test (15%). The validation set is used to check NN generalization. This affects the NN training so that this last one is halted when generalization stops improving, *i.e.*, when the related performance index stops improving. The test set is used to test NN both during and after training, but it has no effect on training. NN performance in training, validation and test is measured by two parameters whose values are in Table 2: the Mean Squared Error (MSE), *i.e.*, the average squared difference between outputs and targets, the Regression (R), which measures the correlation between outputs and targets. Low MSE values and high R values indicate good NN performance.

**Figure 8.**
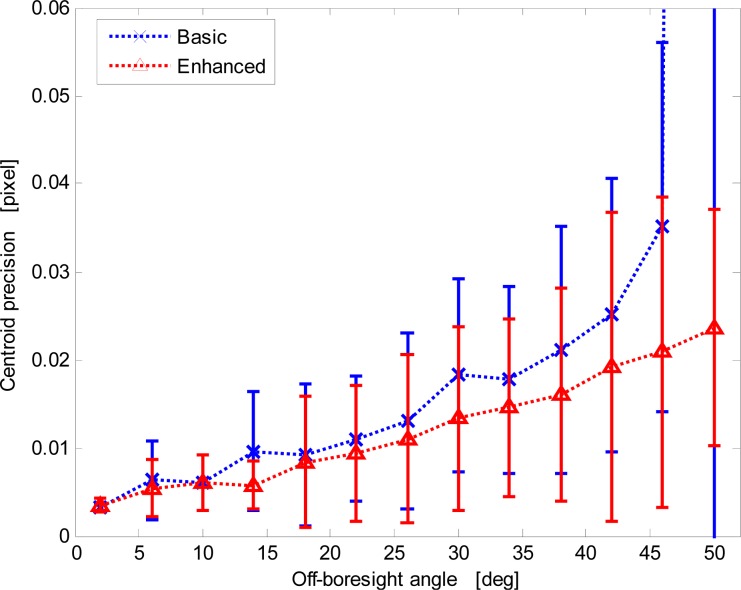
Centroid precision *versus* the off-boresight angle for the basic and enhanced procedures.

Since the average centroid position is used for sun-line computation, sensor measurement performance is firstly assessed in terms of the fluctuations of average centroid position for given illumination direction. Figure 8 shows a well-marked correlation between the average centroid local precision and the illumination direction off-boresight angle for both procedures. In fact, during X-FOV operation the sensor exploits a variable number of imaged sun spots, larger for illuminations close to the boresight and lower for increasing off-boresight angles. Hence, larger off-boresight angles determine worse accuracy. In Figure 8 data are presented as error bars reporting average precision over four-degree intervals of the off-boresight angle and the relevant dispersion in the same intervals.

The enhanced procedure always shows better precision. In addition, it limits the nominal degradation trend even at very large off-boresight angles. Differently, the basic procedure shows a dramatic loss of performance at large off-boresight angles. This is determined by the failure of the basic procedure in detecting the correct size of the array of imaged spots.

**Figure 9.**
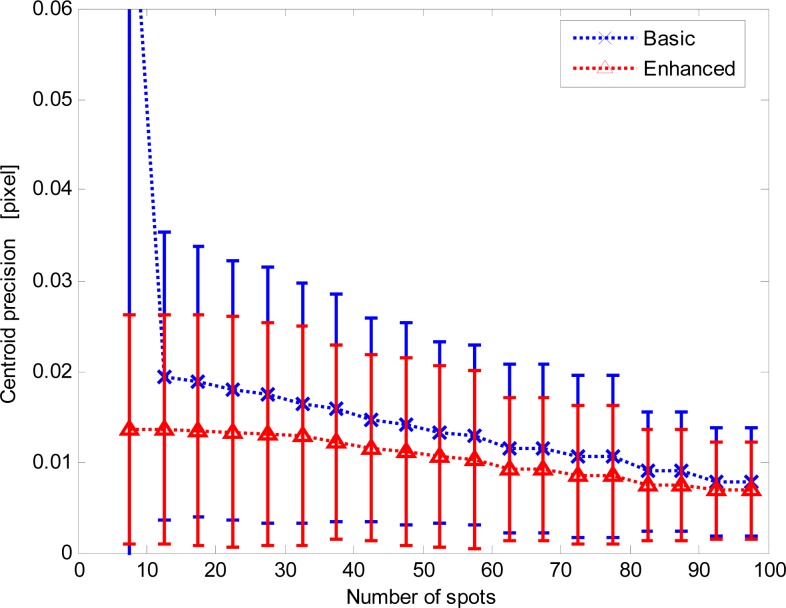
Centroid precision *versus* the number of spots for the basic and enhanced procedures.

To get further insight in this behavior, the local precision data were grouped and averaged according to the number of imaged spots (data are binned over five-spot intervals) detected and exploited to compute the average centroid. Figure 9 shows the centroid local precision average values and standard deviations: the previous results are confirmed.

**Figure 10.**
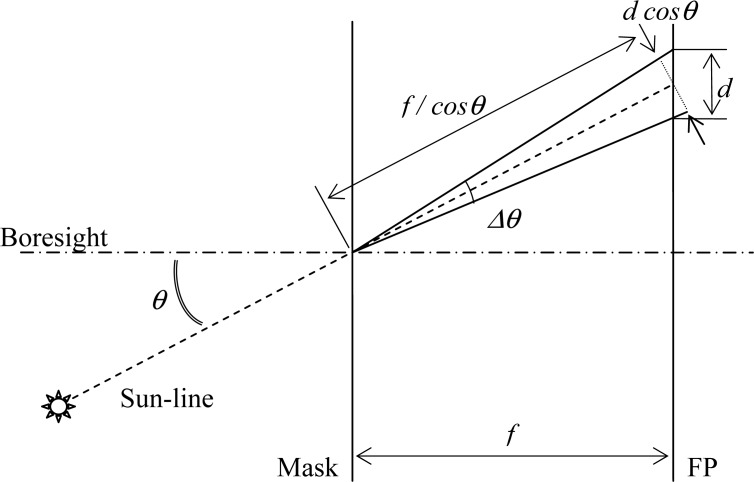
Simplified geometrical model for angular precision prediction.

A prediction of the angular precision can be performed by transforming the centroid-position-based data into angular data. This required some care, because the relation between the fluctuations of the average centroid (*d*) and of the off-boresight angle (*Δθ*) depends on the off-boresight angle; in addition it is not linear due to the imaging geometry. With reference to the simple model displayed in Figure 10 we have:

(3) $$\text{tan}\frac{\Delta \theta }{2}=\frac{\left(d\text{cos}\theta \right)/2}{f/\text{cos}\theta }=\frac{1}{2}\frac{d}{f}{\text{cos}}^{2}\theta$$

where *θ* is the off-boresight angle and *f* is the sensor focal length. The centroid fluctuation is assumed to lie along the radius from the FOV centre to the considered position. This simple one-dimensional model can effectively describe the off-boresight angle effect, thus providing meaningful information on the sensor angular precision.

**Figure 11.**
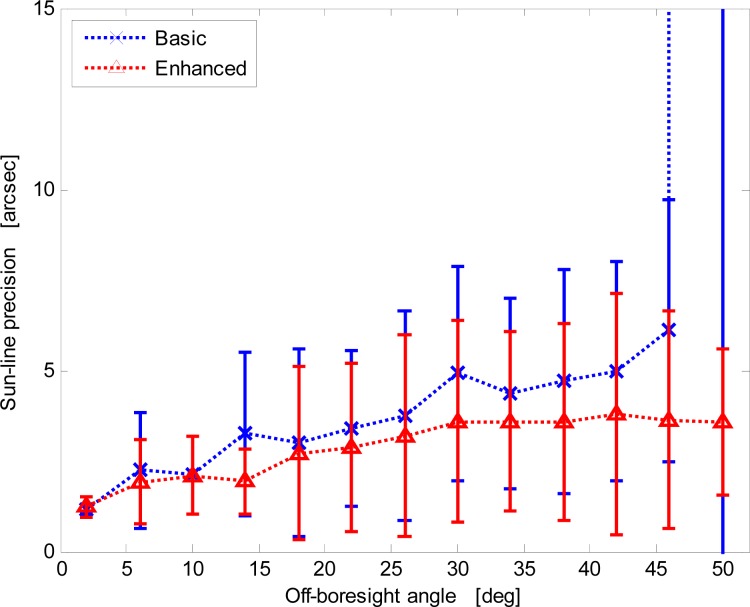
Sun-line predicted precision *versus* the off-boresight angle for the basic and enhanced procedures.

Results are displayed in Figure 11. For the enhanced procedure the trend is almost linear, with the angular precision ranging from about 1 arcsec at boresight to about 4 arcsec at FOV borders (50° off-boresight angle). Differently, with the basic procedure the angular precision exponentially degrades.

**Figure 12.**
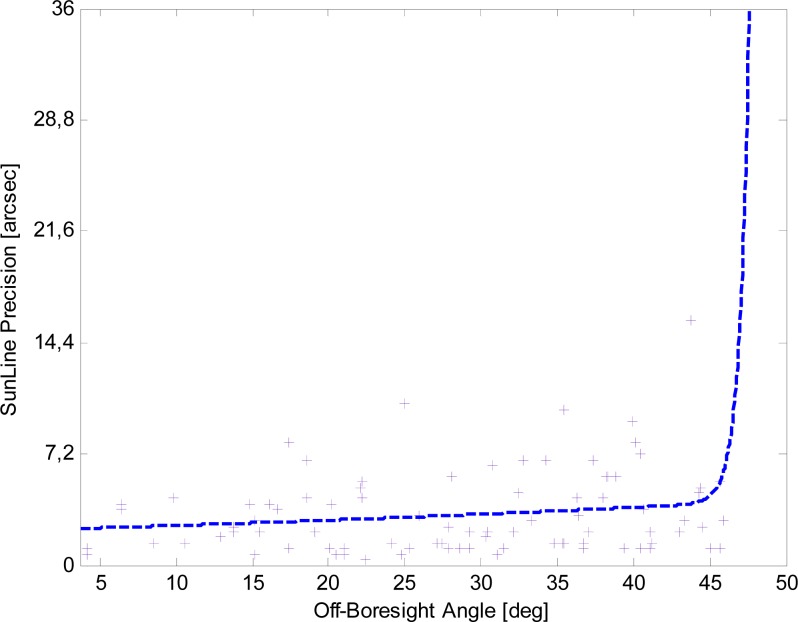
Sun-line precision *versus* the off-boresight angle for the basic procedure.

**Figure 13.**
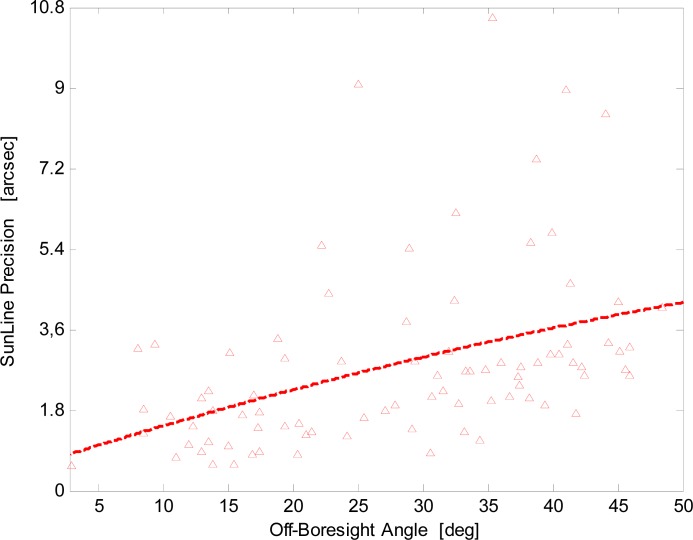
Sun-line precision *versus* the off-boresight angle for the enhanced procedure.

For a more quantitative analysis of the sun-line measurement performance the NN calibration is implemented to transform the average centroid coordinates into the sun-line orientation. The results in Figures 12 and 13 confirm both the overall trend already noticed in the average centroid precision and the angular precision improvement achievable with the enhanced procedure. More specifically, the dashed line in Figure 12 describes the overall trend of the test set sample data, reflecting the average behavior of the basic procedure angular precision of Figure 11 and confirming the steep degradation at large off-boresight angles. It is worth noting that in Figure 12 the y-axis scale has been selected so to clearly observe the angular precision trend as a function of the off-boresight angle. As a consequence, test set sample data determining the steep-degradation behavior in off-boresight positions are not visible in the Figure.

The analysis of Figure 13 confirms that the enhanced procedure significantly improves the sun-line precision: it ranges from about 1 arcsec near the sensor boresight to about 4 arcsec at the X-FOV border. The dashed line in Figure 13 describes the data overall trend that is in good agreement with the average behavior of Figure 11. It is worth noting that sensor accuracy, which does not depend on the off-boresight angle, has an average value of less than 10 arcsec.

## 5. Conclusions

The latest upgrades and tests of the multi-spot micro-sun-sensor under development at the University on Naples were presented. In particular, main focus was on the test of the sensor operation with a variable number of imaged sun spots, which allows sensor operation on a wider FOV. To this end, two issues were carefully investigated: the improvement of the image acquisition procedure, to prevent failures in the identification of the correct number of imaged sun spots near the FOV borders, and the set up of a neural calibration function for the sun-line determination with a variable number of spots. The former one consisted mainly in the introduction of an adaptive shutter time to prevent an excessive image SNR degradation at large off-boresight angles. This new procedure allowed to completely remove previously exhibited failures in sun spot identification. Concerning sensor calibration, a single Neural Network was used to transform the average centroid coordinates on the focal plane into the two angles defining the sun-line orientation.

The improvements produced by the new image acquisition procedure and neural calibration were evaluated during a test campaign conducted with the available sensor model and laboratory test facility. The sun spot image SNR and the precision of the average centroid position resulted significantly higher. Moreover, the exponential degradation of the sun-line measurement precision at FOV border was prevented: high precision (about 4 arcsec) is maintained also at large off-boresight positions, in which it resulted very poor with the procedures formerly in use. These results allow to significantly improve the reliability and precision of the sensor operation with a variable number of spots (*i.e.*, on a wider FOV), so that the advantage of having multiple apertures can be fully exploited.

## Figures and Tables

**Table 1.** Sensor Prototype Relevant Parameters.

**Sensor**	

Focal length (mask-photodetector surface distance)	3 mm
Field of view (FOV):	
Fixed-Spot Mode	±17° × ±8° (10 × 10 spots sun array)
Variable-Spot Mode	±45° × ±40° (3 × 3-spot image at FOV corners)

**Mask**	

Material	steel
Thickness	0.1 mm
Number of holes	100
Hole diameter	0.2 mm
Hole arrangement	10 × 10 array, 0.42 mm pitch (both directions)

**Photodetector**	

Technology	CMOS Active Pixel Sensor
Model	MT 9M001 by Micron Technology, Inc.
Resolution	1280 × 1024 pixels
Pixel size	5.2 × 5.2 μm
Sensing area size	6.66 × 5.32 mm

**Table 2.** NN Performance.

	**MSE(°)**		**R**
Training	1.773 × 10^−4^	∼1	
Validation	6.683 × 10^−4^	∼1	
Test	5.406 × 10^−4^	∼1	
